# LB12. Exebacase Shows Rapid Symptom Resolution in a Phase 2 Study in Adult Patients with *Staphylococcus aureus* bacteremia

**DOI:** 10.1093/ofid/ofab466.1648

**Published:** 2021-12-04

**Authors:** Cara Cassino, Anita F Das, Joy Lipka

**Affiliations:** 1 ContraFect Corporation, Yonkers, NY; 2 AD Stat Consulting, Guerneville, CA; 3 Lipka Consulting, Mullica Hill, New Jersey

## Abstract

**Background:**

Exebacase (EXB), a recombinantly-produced lysin (cell wall hydrolase), is the first direct lytic agent to advance into Phase 3 of clinical development for the treatment of bacteremia including infective endocarditis due to *Staphylococcus aureus*. The microbiologic attributes of EXB, including pathogen-targeted rapid bacteriolysis, and biofilm eradication are distinct from and synergistic with those of traditional antibiotics and underpin the therapeutic potential for EXB.

**Methods:**

The Phase 2 trial was a randomized, double-blind, placebo-controlled multinational study. Patients were randomized (2:1) to receive a single 2-hour infusion of EXB or placebo (PBO) in addition to standard of care antibiotics. Time to resolution of symptoms (shortness of breath, sweating, fatigue and confusion) attributable to the bacteremia was analyzed using Kaplan-Meier methods. Time to resolution was defined as the number of days until all attributable symptoms were absent. If a new (not present at baseline) attributable symptom was present before the baseline symptoms resolved, this new symptom also had to be absent for symptoms to be considered resolved.

**Results:**

A total of 86 patients (53 EXB and 33 PBO) had at least one attributable symptom present at baseline. Of these, symptoms resolved in 94.3% and 87.9% of EXB and PBO patients, respectively. The median time to resolution was 3 days for EXB and 6 days for PBO patients. Median days to symptom resolution in the MRSA group was 3 and 7 days for EXB and PBO patients, respectively, and 3 and 5 days for EXB and PBO patients in the MSSA group, respectively. Time to symptom resolution in MRSA patients in presented in Figure 1.

Figure 1. Time to Resolution of Symptoms in Patients with MRSA Bacteremia including Infective Endocarditis

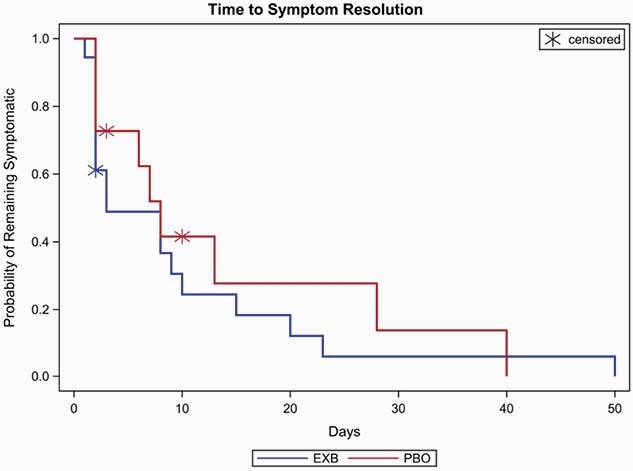

**Conclusion:**

The majority of EXB and PBO patients had symptom resolution. However, EXE patients achieved symptom resolution in 3 days compared with 6 days for PBO patients overall, and 7 days for PBO patients with MRSA. These data suggest that rapid bacteriolysis may translate to a clinical benefit for patients receiving EXB and aligns with a median length of hospital stay of 6 and 10 days among US MRSA patients that received EXE and PBO patients, respectively. (Fowler, et al, 2020).

**Disclosures:**

**Cara Cassino, MD**, **ContraFect Corporation** (Employee) **Anita F. Das, PhD**, **Adagio** (Consultant)**AN2** (Consultant)**Cidara** (Consultant)**ContraFect** (Consultant)**Iterum** (Consultant)**MicuRx** (Consultant)**Paratek** (Consultant)**Union** (Consultant) **Joy Lipka, MS**, **ContraFect Corporation** (Consultant)

